# Effects of Different Levels of Flea Infestation on Gut Microbiota of Brandt’s Voles (*Lasiopodomys brandtii*) in China

**DOI:** 10.3390/ani15050669

**Published:** 2025-02-25

**Authors:** Zhenxu Wang, Lu Wang, Chenran Guo, Zihao Wang, Xinchang Lun, Haoqiang Ji, Meng Shang, Xiaoxu Wang, Qiyong Liu

**Affiliations:** 1 National Key Laboratory of Intelligent Tracking and Forecasting for Infectious Diseases, National Institute for Communicable Disease Control and Prevention, Chinese Center for Disease Control and Prevention, Beijing 102206, China; wangzhenxuicdc@163.com (Z.W.); lxc960506@163.com (X.L.); 2 School of Public Health, Cheeloo College of Medicine, Shandong University, Jinan 250012, China; wanglu8771@163.com (L.W.); jihaoqiang666@mail.sdu.edu.cn (H.J.); m_shang111@163.com (M.S.); 15232236121@163.com (X.W.); 3 School of Public Health, Nanjing Medical University, Nanjing 211166, China; 18855195628@163.com (C.G.); dyw_i525.1999@foxmail.com (Z.W.)

**Keywords:** parasite–host interaction, immune response, microbial diversity, 16S rRNA sequencing, metabolism and development

## Abstract

Brandt’s vole is a common small rodent, and its gut microbiota is critical to host health and immune function. The parasitic fleas commonly found in Brandt’s voles cause an immune response, but their impact on the gut microbiota remains unclear. According to the level of flea infestation, Brandt’s voles were divided into the control group, low-infestation group, and high-infestation group. The changes in the microbial community composition, abundance, and diversity of the gut microbiota were evaluated using 16S rRNA sequencing. Flea infestation significantly affected body weight, food intake, and gut microbiota structure. The low-infestation group exhibited the most pronounced changes in weight and food intake, while the high-infestation group showed the least. In the 4th week, 16S rRNA sequencing revealed an increase in alpha diversity and alterations in microbial composition. Beta-diversity analysis indicated significant differences in the intestinal microbiota between the experimental groups and the control group. By the 8th week, these differences had diminished, suggesting that the microbiota had stabilized or recovered over time. Overall, parasitic flea infestation significantly alters the diversity, structure, and characteristic microbial enrichment of the gut microbiota in Brandt’s voles, potentially impacting host metabolism, immunity, and growth. While this study lasted 8 weeks, the long-term health effects of flea infestation may persist. Future research should elucidate the interaction mechanisms between parasites and hosts, define the time frames and mechanisms of these long-term impacts, and provide theoretical support for animal health management and disease control.

## Simple Summary

This study investigated the effects of parasitic flea infestation on the intestinal microbiota of Brandt’s voles. The voles were divided into the control group, low-infestation group, and high-infestation group, and changes in the intestinal microbiota were evaluated by 16S rRNA sequencing. The results indicated that flea infestation affected body weight, food intake, and intestinal microbial community structure, with the most significant changes in the low-infestation group. In the early stage (4th week), alpha diversity increased and microbial composition shifted, while beta-diversity analysis revealed differences between the experimental and control groups. By the 8th week, these differences had diminished, suggesting that the microbiota had stabilized or returned to a baseline state. This study highlights the long-term effects of flea infestation on the gut microbiome, metabolism, and development in voles, but it only lasted for 8 weeks.

## 1. Introduction

Plague is one of the most dangerous zoonotic infestations in Asia. It is a severe infectious disease caused by the bacterium *Yersinia pestis* (Yersin, 1894), characterized by acute onset, strong infectivity, and high mortality [1,2]. In recent years, the natural foci of *Yersinia pestis* in the Inner Mongolia Autonomous Region have become increasingly active, with a total of three cases of plague reported between 2012 and 2021. Additionally, from 2017 to 2020, *Yersinia pestis* was isolated from fleas parasitizing rodents for four consecutive years [3]. Fleas, as vectors for various pathogens, primarily spread diseases by biting host animals and subsequently transmitting the pathogens to humans. The occurrence and spread of flea-borne diseases pose significant risks to human health and safety [4].

In recent years, various types of symbiotic relationships between small mammals and representatives of different classes of animals have been known and studied [5]. Fleas, in particular, have a close symbiotic relationship with rodents. These ectoparasites live on the surfaces of their hosts, relying on them for nutrition [6]. As a result, fleas primarily interact with the host’s epidermis and can trigger behavioral defense responses. Both behavioral defense and immune defense require significant energy costs, and the host’s energy expenditure is positively correlated with the external parasite load [7]. Studies have shown that C57BL/6 mice exhibit an immune response to flea bites, which is characterized by mild inflammation, the aggregation of neutrophils and macrophages at the bite site, and the production of IgG1 antibodies [8], although the levels of this response can vary among individuals. However, this response may vary from species to species and among individuals and should not be generalized as a common phenomenon in all rodents. Furthermore, flea infestation and flea allergy are two different concepts. The former mainly involves the host’s immune defense mechanism, while the latter is an allergic reaction that usually occurs in a small number of individuals.

The gut and its microbiota form a unique ecosystem in mammals, with microorganisms playing a vital role in maintaining host health. The disruption of this balance is usually caused by external stimuli and is harmful to both the microbiome and the host [9]. For instance, studies have shown that parasitic infestation, such as with *Toxoplasma gondii* in BALB/C mice, can alter the relative abundance and diversity of the gut microbiota [10].

Research on gut microbiota typically focuses on host metabolism, digestion, immunity, and behavior [11]. Recently, Wang’s team provided important insights into how the gut microbiota of Brandt’s voles adapts to cold environments, influencing metabolic needs, thermoregulation, and cognitive behavior [12,13,14,15]. In addition, the effects of internal parasites on rodent gut microbiota have been explored. For example, Rapin A’s study found that polyhedra infestation in the small intestines of mice led to significant changes in the microbial communities in both the small and large intestines of wild-type C57BL/6 mice [16]. Similarly, Wang ZH et al. demonstrated that flea parasitism can alter the composition and diversity of gut microbiota in Mongolian gerbils in both natural and laboratory settings [17]. However, there are few studies that control for varying intensities of flea infestation to investigate how flea parasitism affects the gut microbiota composition and diversity in the host. We hypothesize that flea parasitism intensity modulates the gut microbiota composition and diversity in the host, thereby influencing host metabolism and overall health. This study aims to explore the interaction mechanism between flea parasitism and host physiology by examining changes in gut microbial community structure and host metabolic profiles. Our findings will provide new insights into how parasite infestation regulates the physiological state of the host and offer a theoretical basis for improving animal health management and developing prevention and control strategies for related diseases.

## 2. Materials and Methods

### 2.1. Study Animals

The experimental animals were derived from a breeding population of Brandt’s voles maintained in the laboratory of the Experimental Animal Center at the Chinese Center for Disease Control and Prevention. Fifteen male Brandt’s voles, aged 5–7 months (usually still growing but close to full maturity) and weighing approximately 50 g, were selected for this study. The animals were housed in a controlled environment with a temperature of 22 ± 1 °C, humidity of 65 ± 5%, and a 14 h light/10 h dark photoperiod. Two weeks of acclimatization was allowed before the start of the experiment. Each vole was housed in an individual cage (30 × 30 cm) with corncobs as nesting material, which were replaced regularly. The animals were provided with rabbit breeding feed (Beijing Keao Xieli Feed Co., Ltd., Beijing, China) and had free access to food and water. The experimental feed was provided by Beijing Ke’ao Xieli Feed Co., Ltd., and designed specifically for the growth and reproduction of rodents. The main components and nutritional composition of the feed are as follows: Main ingredients: corn, soybean meal, alfalfa powder, salt, calcium hydrogen phosphate, stone powder, various vitamins, various trace elements, etc. Nutritional composition: Crude Protein ≥ 170 g; Crude Fat ≥ 30 g; Crude Fiber: 100–150 g; Lysine ≥ 8 g; Egg + cysteine ≥ 6 g; Arginine ≥ 8 g; Tryptophan ≥ 2.7 g; Histidine ≥ 3.5 g; Phenylpropanoid + tyrosine ≥ 13.0 g; Threonine ≥ 6.5 g; Leucine ≥ 13.0 g; Isoleucine ≥ 7.2 g; Valine ≥ 8.3 g; moisture content ≤ 110 g; crude ash content ≤ 90 g, etc.

The fleas used in the study were sourced from our laboratory colony. Each vole was inoculated with *Xenopsylla cheopis* and placed in a glass tank (separate for each animal). The tanks were lined with a combination of wood chips and corn chips to create a suitable refuge for the fleas. The researchers involved in the study held valid laboratory animal practitioner certificates, and all procedures were approved by the Laboratory Animal Welfare and Ethics Review Committee of the Chinese Center for Disease Control and Prevention.

### 2.2. Experimental Design

Before flea inoculation, the Brandt’s voles were weighed. The 15 male voles were randomly assigned to one of three groups, with 5 animals in each group: a control group (0 fleas/mouse), a low-infestation group (20 fleas/mouse), and a high-infestation group (50 fleas/mouse). Each group was housed separately under the same environmental conditions for 8 weeks. Weekly measurements were taken for body weight and food intake (as indicators of the voles’ physiological response to flea infestation). In weeks 4 and 8 of the experiment, we placed the mice in clean, sterile containers or cages and collected fecal particles when they were naturally excreted. After collection, the fecal samples were processed immediately to minimize microbial activity and maintain sample integrity. Each fecal sample (usually 100–200 milligrams) was transferred to a sterile Eppendorf tube. The sample was immediately frozen in liquid nitrogen for 15 min and then stored at −80 °C until further analysis. This rapid freezing and storage solution helps prevent microbial metabolic activity and maintain the composition of the microbial community.

### 2.3. 16S rRNA Gene Amplicon Sequencing and Bioinformatics Analysis

The NEBNext ^®^ Ultra DNA Library Prep Kit was used to prepare the DNA library, and the quality was evaluated before sequencing on the Nova Seq 6000 platform. QIIME2 version 2024.10 software (quantitative study of microbial ecology 2) was used to analyze the 16S rRNA gene sequence and was provided by Noho Bioinformation Technology Co., Ltd. (Beijing, China). The microbial community composition was analyzed by various statistical methods, such as ANCOM (Analysis of Composition of Microbiomes), ANOVA, Kruskal–Wallis test (K-W test), and LEfSe (linear discriminant analysis). The overlap of amplicon sequence variations (ASVs) between groups was visualized using a Venn plot. Alpha-diversity indices, such as Observed ASVs, Chao1, Shannon–Wiener, Simpson, and Faith’s Phylogenetic Diversity Index, were calculated to assess the richness and evenness of the samples, with higher values indicating greater diversity. Beta diversity, comparing microbial community compositions across samples, was analyzed using principal coordinate analysis (PCoA) based on the Jaccard distance. LEfSe was also used to identify characteristic microbes that differentiated between groups and to assess the magnitude of these differences.

### 2.4. Statistical Analyses

All data were tested by a normal distribution test (Shapiro–Wilk test) and homogeneity of variance test (F test). Excel 2019 software and SAS 9.4 software were used for statistical analysis and mapping. Variance analysis was used to compare the body weight, food intake, and the number of surviving fleas on Brandt’s voles in each group in the last week of the trial. The body weight change and food intake of Brandt’s voles were compared by a generalized estimating equation (GEE). In 16S rRNA amplicon analysis, the Kruskal–Wallis method was used to compare the difference in alpha diversity, and similarity analysis (ANOSIM) was used to compare the difference in beta diversity. The results are expressed as mean ± standard error (mean ± SE). *p* < 0.05 indicated that the difference was statistically significant.

## 3. Results

### 3.1. Changes in Average Body Weight and Food Intake of Brandt’s Voles and Number of Surviving Fleas

**Figure 1:**
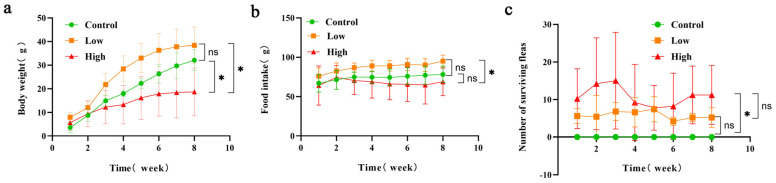
(**a**) Changes in the average body weight of Brandt’s voles over an 8-week infestation period in the control group, the low-infestation group, and the high-infestation group. (**b**) Changes in the food intake of Brandt’s voles over an 8-week infestation period in the control group, the low-infestation group, and the high-infestation group. ^ns^ *p* > 0.05; * *p* < 0.05. (**c**) Changes in the average number of surviving fleas of Brandt’s voles over an 8-week infestation period in the control group, the low-infestation group, and the high-infestation group.

As depicted in Figure 1a, the overall trend in body weight changes in Brandt’s voles is upward. When comparing the control group, the low-infestation group, and the high-infestation group, the low-infestation group exhibited the most significant increase in body weight, while the high-infestation group showed the smallest change. Throughout the entire experimental period, both the control group and the low-infestation group demonstrated consistent upward trends in body weight. In contrast, the body weight of Brandt’s voles in the high-infestation group initially increased during the first 0–6 weeks but then stabilized between weeks 6 and 8. We compared the differences in average weight changes between the groups in the 8th week. The results of analysis of variance showed that the weight gain of the high-infestation group was significantly lower than that of the control group, and there was also a significant difference between the low-infestation group and the high-infestation group, indicating that high-level flea infestation significantly limits the host’s weight gain (*p* < 0.05).

As shown in Figure 1b, the food intake of the Brandt’s voles exhibits a dynamic pattern over time. During the first 1–8 weeks, the food intake in both the control group and the low-infestation group initially increased and then stabilized. In contrast, during weeks 1–7, the food intake of the highly infested group first increased and then decreased, while during weeks 7–8, the food intake of the highly infested group began to increase. This indicates that the impact of infestation on food intake is staged, which may be related to the host’s immune response, energy requirements, and the severity of the infestation. During 7–8 weeks, an increase in food intake may reflect the host’s adaptive response to infestation, as the host maintains its energy balance by regulating food intake. According to the results of variance analysis of the average food intake between the groups, in the 8th week, we found that the food intake of the low-infestation group was significantly higher than that of the high-infestation group (*p* < 0.05).

As shown in Figure 1c, we compared the number of surviving fleas between the control group, the low-infestation group, and the high-infestation group in the 8th week. The variance results showed that there was a statistically significant difference between the control group and the high-infestation group (*p* < 0.05). The number of surviving fleas in the high-infestation group was significantly lower than that in the control group, indicating that strong infestation may have a significant negative impact on the survival of fleas. The number of surviving fleas in the low-infestation group was similar to that in the control group, which may indicate that low-level infestation did not have a significant effect on the survival of fleas.

The effects of flea parasitism on the body weight of Brandt’s voles were examined using generalized estimating equations (GEEs). In this analysis, body weight was designated as the response variable, while group assignment served as the independent variable. Additionally, food intake and time (measured in weeks) were included as covariates. The model’s effect test, as presented in Table 1, revealed that flea infestation significantly influenced weight change (*p* < 0.05). Similarly, both time and food intake had significant effects on weight change (*p* < 0.05).

### 3.2. Results of 16S rRNA Sequencing

A Venn diagram was constructed based on the number of ASV (amplicon sequence variant) samples to visually illustrate the similarity and overlap in microbial composition between groups. The results showed that in the 4th week, there were 822 ASVs unique to the control group and 908 ASVs unique to the low-infestation group, with a total of 904 ASVs in both groups. In the high-infestation group, there were 782 ASVs unique to the control group and 786 ASVs unique to the high-infestation group, with a total of 1044 ASVs shared between them (Figure S1a,b). In the 8th week, the control group had 869 unique ASVs, the low-infestation group had 882 unique ASVs, and the two groups shared 990 ASVs. In the high-infestation group, the control group had 837 unique ASVs, the high-infestation group had 855 unique ASVs, and the two groups shared 1022 ASVs (Figure S1c,d). From the graph, it can be seen that as the sample size of the gut microbiota of Brandt’s voles increases, the dilution curve tends to flatten out, showing a clear asymptotic line and indicating a sufficient sequencing volume. This suggests that the sampling of the gut microbiota of Brandt’s voles in this study is close to complete and can represent the species diversity in the samples (Figure S2a,b).

### 3.3. Analysis of Species Composition and Differences in Brandt’s Voles

**Figure 2:**
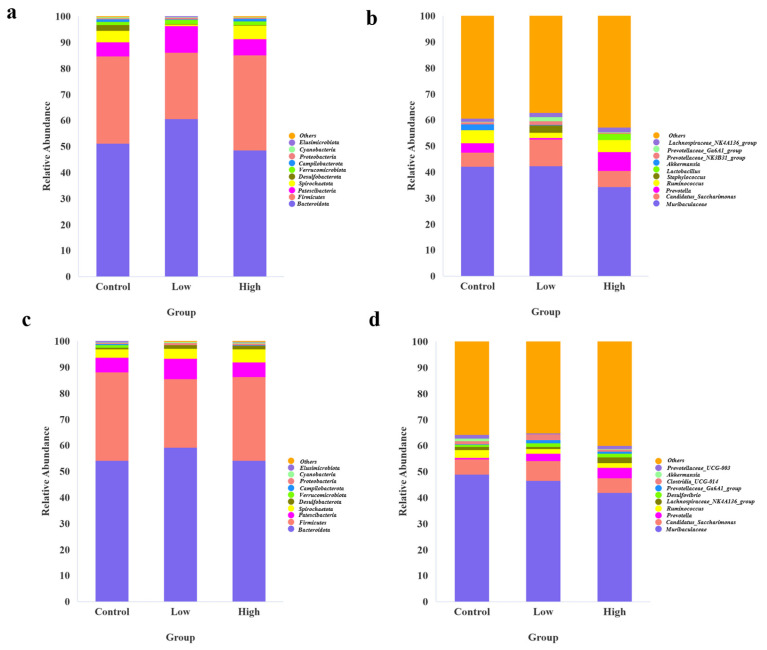
(**a**) A stacked distribution diagram of gut microbiota species composition at the phylum level among the control group, low-infestation group, and high-infestation group in the 4th week. (**b**) A stacked distribution diagram of gut microbiota species composition at the genus level among the control group, low-infestation group, and high-infestation group in the 4th week. (**c**) A stacked distribution diagram of gut microbiota species composition at the phylum level among the control group, low-infestation group, and high-infestation group in the 8th week. (**d**) A stacked distribution diagram of gut microbiota species composition at the genus level among the control group, low-infestation group, and high-infestation group in the 8th week.

As shown in Figure 2a,c, at the phylum level, the two bacteria with the highest relative abundance across the control group, low-infestation group, and high-infestation group in the 4th and 8th weeks are *Bacteroidota* and *Firmicutes*, respectively.

We calculated the ratio of these two bacteria separately and found that in the 4th week, the B/F (*Bacteroidota*/*Firmicutes*) ratio was highest in the low-infestation group, followed by the control group, and lowest in the high-infestation group. In the 8th week, the B/F ratio in the low-infestation group remained the highest, but the B/F value of the high-infestation group increased, while the B/F value of the control group decreased to the lowest. This result reveals that the low-infestation group of Brandt’s voles may have maintained a healthier and relatively balanced gut microbiota through a lower parasite burden, with *Bacteroidota* dominating in this environment, resulting in the highest B/F ratios. In contrast, in the high-infestation group, a higher parasite burden may trigger a stronger immune response that inhibits the growth of some beneficial bacteria (such as *Bacteroides*), leading to the lowest B/F ratio. The control group, which was not affected by parasites, exhibited a relatively fragile gut microbiota structure, resulting in the lowest B/F ratio among all groups. At the genus level, the two bacteria with the highest relative abundance across the control group, low-infestation group, and high-infestation group in both the 4th and 8th weeks were *Muribaculaceae* and *Candidatus_Saccharimonas*, respectively (Figure 2b,d).

### 3.4. Alpha-Diversity Analysis

**Figure 3:**
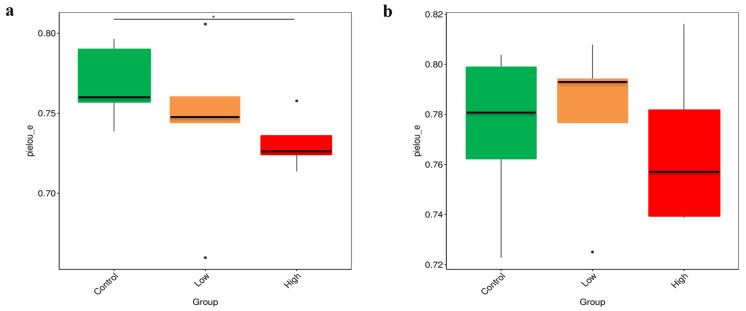
(**a**) Differences in the Pielou_e index among the control group, the low-infestation group, and the high-infestation group in the 4th week. (**b**) Differences in the Pielou_e index among the control group, the low-infestation group, and the high-infestation group in the 8th week. * *p* < 0.05.

The results of alpha-diversity analysis revealed that, in the 4th week, the Pielou’s evenness (Pielou_e) index of Brandt’s voles in the control group was significantly higher than that in the high-infestation group (*p* < 0.05) (Figure 3a). In the 8th week, there was no difference in the Pielou_e index among the control group, the low-infestation group, and the high-infestation group (Figure 3b). The significant difference in the Pielou_e index between the control and high-infestation groups in the 4th week may be attributed to the imbalance in gut microbiota caused by the high parasite burden. This imbalance likely led to the dominance of certain microbial taxa, resulting in an uneven microbial community structure. In contrast, in the 8th week, the lack of significant differences in the Pielou_e index across all groups suggests that the host’s adaptability had improved, immune responses had been regulated, and the gut microbiota had recovered. These changes collectively contributed to a more balanced microbial evenness among the groups.

### 3.5. Beta-Diversity Analysis

In the 4th week of the experiment (Figure S3a), beta-diversity analysis based on the Jaccard distance revealed significant differences between the control group and both the low-infestation group and the high-infestation group (*p* < 0.05). However, by the 8th week, Jaccard distance analysis showed no significant differences among all groups (Figure S3b). Overall, in the 4th week, the degree of infestation (both low- and high-infestation groups) had a significant impact on the diversity of gut microbiota in Brandt’s voles. This effect may be attributed to flea infestation, which altered the microbial environment within the host. By the 8th week, despite the persistence of infestation differences, the gut microbiota diversity had returned to a more stable state, likely due to the passage of time, adaptive changes, or other external factors.

**Figure 4:**
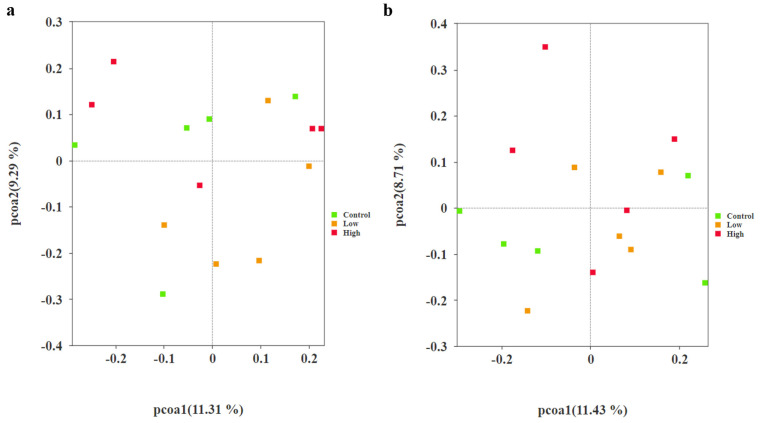
(**a**) The microbial community structure of the PCoA plot based on the Jaccard distance among the control group, the low-infestation group, and the high-infestation group in the 4th week. (**b**) The microbial community structure of the PCoA plot based on the Jaccard distance among the control group, the low-infestation group, and the high-infestation group in the 8th week.

Principal component analysis (PCoA) was performed on the samples using the Jaccard distance algorithm for both the 4th and 8th weeks, respectively (Figure 4a,b). The results indicated that the explanatory powers of the principal coordinate axes PCoA1 and PCoA2 were 11.31%, 9.29%, 11.43%, and 8.71%, respectively. PERMANOVA analysis was used to compare differences in gut microbial composition between groups, revealing no significant differences in gut microbiota structure (*p* > 0.05). This suggests that there were minimal differences in spatial distribution between microbial communities across the groups.

### 3.6. LEfSe Analysis

**Figure 5:**
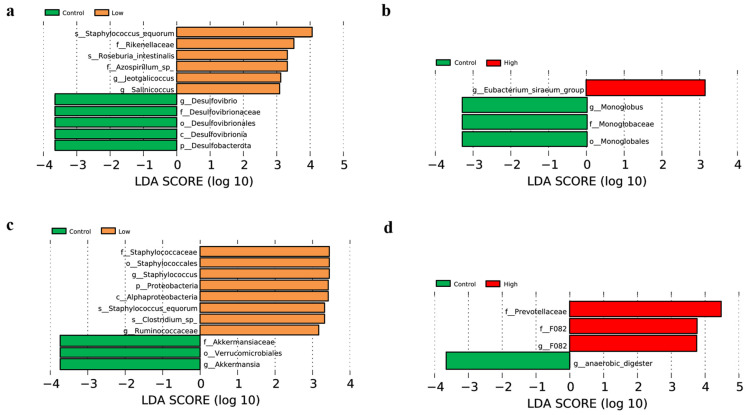
(**a**) The results of LEfSe analysis between the control group and the low-infestation group in the 4th week. (**b**) The results of LEfSe analysis between the control group and the high-infestation group in the 4th week. (**c**) The results of LEfSe analysis between the control group and the low-infestation group in the 4th week. (**d**) The results of LEfSe analysis between the control group and the high-infestation group in the 8th week.

LEfSe analysis identified characteristic microbes for each group, with each horizontal bar representing a specific microbial species. The length of the bar indicates the corresponding LDA (linear discriminant analysis) value, and a threshold LDA value of greater than 2 was used to identify significant biomarkers. Longer bars signify higher enrichment levels of the respective microbes. In the 4th week, comparisons between the control group and the low-infestation group, as well as between the control group and the high-infestation group, revealed the following biomarker distributions: the control group had five and three enriched biomarkers, respectively; the low-infestation group had six enriched biomarkers; and the high-infestation group had only one enriched biomarker (Figure 5a,b). By the 8th week, similar comparisons showed that the control group had three and one enriched biomarkers, respectively; the low-infestation group had eight enriched biomarkers; and the high-infestation group had three enriched biomarkers (Figure 5c,d).

## 4. Discussion

Our research demonstrates that with varying levels of flea infestation, the weight and food intake of Brandt’s voles undergo significant changes. Specifically, weight changes across different groups exhibit dynamic trends. Given that the Brandt’s voles in this study were 5–7 months old and still in their growth and development stage, the weight gain of the control group and the high-infestation group initially increased before stabilizing. However, the weight gain in the high-infestation group was significantly lower than that of the control group, suggesting that a high level of flea infestation limits the host’s weight gain. This finding aligns with the study by Wang et al. [17] on flea infestation in Mongolian sand gerbils, which suggests that a higher parasitic burden may severely impact the host’s nutrient uptake [18]. Interestingly, our study also revealed that the weight of Brandt’s voles in the low-infestation group continued to increase, suggesting that low-level flea infestation may actually promote host growth and development, leading to larger body size compared to the age-matched control group. Khokhlova et al. observed, in their study of fleas and sand rats, that even mild flea stress can stimulate the host’s immune response [19,20]. In addition, Devereaux et al. observed that small hamsters bitten by fleas experienced a decrease in body weight and length, a decrease in hematocrit levels, and an increase in the resting metabolic rate [21]. In terms of feeding behavior, the food intake of different groups dynamically changed over time. At first, the food intake of each group increased, but in the middle and later stages of flea infestation, the food intake of the control group tended to stabilize. However, the food intake of the low-infestation group continued to increase, while the food intake of the high-infestation group decreased over time. Regarding the improvement trend in weight gain and feed intake between the low-infestation group and the control group, we believe that there may be several explanations. First, low levels of infestation may trigger a modest immune system response, which, in some cases, promotes better metabolism and weight gain. In addition, the low-infestation group may show higher weight gain through physiological adaptation mechanisms or better feed digestion and absorption. However, since this trend does not show significant statistical differences, we believe that these results need more experimental verification to confirm these hypotheses. The weight gain of the high-infestation group was significantly lower than that of the control group. We believe that this may be due to the multiple effects or behaviors of high-level flea infestation on the physiological processes of the host. A high level of flea infestation limits the host’s weight gain, or high-level flea infestation may shift the host’s attention to behaviors such as combing hair, which may reduce food intake by enhancing the immune response, aggravating nutritional loss, inducing a chronic inflammatory response, and possibly affecting the digestive system.

The measurement results of the gut microbiota in the 4th and 8th weeks revealed that parasitic flea infestation significantly altered the gut microbiota of the Brandt’s voles in this study. Notably, in the 4th week, the B/F values were highest in the low-infestation group and lowest in the high-infestation group. The B/F value reflects the ratio of beneficial to harmful bacteria in the intestine, and a higher B/F value in the low-infestation group suggests that parasitic flea infestation may promote the growth of beneficial bacteria. Conversely, with the increase in infestation level, the high-infestation group showed lower B/F values, indicating that high-level parasitic flea infestation may lead to an increase in the relative abundance of harmful bacteria, which in turn may have adverse effects on the health of the host. This phenomenon is consistent with the results of other studies, indicating that parasitic infestations can affect the host’s immune function and metabolic status by altering the structure of the gut microbiota [22]. By the 8th week, the diversity of the gut microbiota appeared to stabilize, suggesting that after prolonged infestation, the host’s gut microbiota adapted to the presence of parasitic fleas to a certain extent and showed a certain degree of recovery. Low-level parasitic flea infections may have some positive effects on hosts, which is consistent with recent research on the complex effects of parasitic infections on host health and microbial communities. For example, some studies suggest that low-level parasitic infections may have a positive impact on host health by regulating the host’s immune system and gut microbiota, promoting the growth of beneficial bacteria [23,24,25]. This phenomenon has also been found in other parasitic studies, such as those on low-level worm infection, which is believed to increase the diversity of intestinal bacteria and may have a protective effect on certain inflammatory diseases. However, the difference between the high-infestation group and the control group remains significant, indicating that parasitic flea infestation may trigger changes in the gut microbiota.

The changes in gut microbiota diversity are particularly noteworthy. By comparing Pielou’s evenness (Pielou_e) index, we found a significant difference between the control group and the high-infestation group in the 4th week. Relative to the control group, the Pielou_e evenness of the high-infestation group was relatively low, indicating an imbalance in the microbial community, with some microorganisms dominating. This imbalance may be caused by the overgrowth of harmful bacteria and the decrease in beneficial bacteria, leading to potential health problems [26,27,28,29]. However, by the 8th week, the differences in the Pielou_e index between groups had disappeared. This suggests that, while parasitic flea infestation significantly impacts gut microbiota in the early stage, the gut microbiota gradually stabilizes over time. This stabilization likely reflects the host’s adaptation to infestation and the subsequent recovery of the gut microbiota balance.

Beta-diversity analysis using the Jaccard distance index revealed significant differences in gut microbiota structure between the low-infestation group and the high-infestation group and the control group in the 4th week. However, no significant differences were observed in the 8th week. This shift may be attributed to the initial, pronounced disruption of the host’s intestinal microbiota by flea infestation. Over time, the host and its intestinal microbiota gradually adapt and recover. In this process, the host’s immune system and microbial community may reach a new equilibrium state through interaction. Similar studies have also found that the host microbial community usually undergoes dynamic changes during long-term parasitic infestation and eventually tends to be stable, but the presence of parasites still has a long-term impact on the host [30].

LEfSe analysis revealed significant differences in the intestinal microbial characteristics among different groups. In both week 4 and week 8, distinct enrichment changes were observed in biomarkers between the control group and the low-infestation group, as well as between the control group and the high-infestation group. The low-infestation group exhibited a high number of enriched biomarkers in week 4 and week 8, indicating that low-level parasitic flea infestation may promote the enrichment of some beneficial microorganisms. In contrast, the high-infestation group showed fewer enriched biomarkers, which may be related to the heavier burden of parasites and the increase in harmful bacteria in the host intestine. This result suggests that the levels of parasite infestation may directly affect the structure and function of the gut microbiota, thus affecting the health of the host to varying degrees. Specifically, the relative abundance of *Eubacterium siraeum* in the high group was higher than that in the control group in the 4th week. Pan et al. found that metabolites secreted by *Eubacterium siraeum* could inhibit weight gain in obese mice by reducing subcutaneous adipocyte size, increasing the number of brown adipocytes, and lowering the levels of leptin, interleukin-6, and insulin [31,32,33]. *Eubacterium siraeum* has also been shown to degrade tyrosine and use it as a carbon and nitrogen source for growth [34,35,36]. The upregulated blood metabolites in our study were significantly enriched in PIP2 hydrolysis, while downregulated metabolites were primarily involved in the PI3K/AKT signaling pathway. This suggests that the blood environment of the voles underwent important physiological changes, with reduced activity of the PI3K/AKT pathway, which in turn could impair lipid synthesis [37,38,39].

However, the results of principal coordinate analysis (PCoA) did not show significant differences, which may be due to significant variations between samples or changes in gut microbiota beyond the capture range of the PCoA method. For example, in some studies, PCoA failed to fully distinguish microbial community differences between different groups, indicating that this method may not be sensitive enough to complex microbial community changes [40]. Similar phenomena have also been observed in studies of other rodents. For example, mice infected with Trichinella spiralis exhibit changes in gut microbiota composition, particularly an increase in the relative abundance of Proteobacteria, while the abundance of *Bacteroidetes* and *Clostridiales* decreases [41]. These results are similar to our research findings, indicating that the impact of parasitic infestations on gut microbiota may have some universality. In addition, studies have shown that parasitic infestations not only directly affect the gut microbiota but also may indirectly impact the microbiota by altering the host’s immune response. For example, certain immunodeficiency diseases (such as CTLA4-D) are associated with specific changes in the gut microbiota [42], which further illustrates the complex interaction between host immune status and gut microbiota. In summary, although PCoA did not detect significant differences in our study, future research is expected to more comprehensively reveal the impact of parasitic infestations on the gut microbiota by combining other statistical methods and more sample data.

This study has several notable limitations: 1. Small sample size: This study utilized only five laboratory-raised rodents, which restricts the statistical power and generalizability of the findings. Laboratory animals, which often exhibit genetic homogeneity and phenotypic uniformity due to inbreeding, may differ significantly from wild populations in terms of genetic background, environmental adaptability, and behavior. Therefore, caution is warranted when extrapolating these results to natural populations. In future work, increasing the sample size and incorporating animals with diverse genetic backgrounds would enhance the representativeness and robustness of the findings. 2. Differences between lab animals and wild populations: Laboratory animals, typically inbred, display high genetic homogeneity and phenotypic uniformity. While this characteristic is beneficial for controlling experimental conditions, it may limit the applicability of the results to wild populations that face complex ecological pressures and parasite infestations. In future work, conducting studies in wild populations could validate the laboratory findings and provide a more realistic ecological context. 3. Short experimental duration: The study’s 8-week observation period is insufficient to capture the long-term effects of flea infestation on host weight, food intake, and microbiota. Long-term infestations may have more complex impacts on the host’s immune system and metabolism, which may not be fully revealed in short-term experiments. In future work, extending the study duration to at least 12 weeks would allow for a more comprehensive understanding of the long-term dynamics of flea infestation. 4. Representativeness of fecal samples: This study did not address whether fecal samples accurately reflect the microbiota in different intestinal regions. Although fecal samples are commonly used, they may not fully represent the microbiota diversity across the gut. In future work, combining fecal sampling with direct sampling from different intestinal regions could provide a more comprehensive understanding of gut microbiota distribution. These limitations underscore the need for further research to address these issues comprehensively.

## 5. Conclusions

In summary, different levels of parasitic flea infestation had a significant effect on the gut microbiota of Brandt’s voles, which was manifested in the diversity of the gut microbiota, the change in flora structure, and the enrichment of characteristic microorganisms. These changes may have an important impact on the host’s metabolism, immune function, and growth and development. Although our experimental period was only 8 weeks, the potential impact of parasitic flea infestation on host health may persist for a longer period of time after the end of the experiment. Future research should explore the interaction mechanisms between parasites and hosts, clarify the specific time frame and potential mechanisms of these long-term effects, and provide a theoretical basis for animal health management and prevention and control strategies for related diseases.

## Figures and Tables

**Table 1:** Model’s effect test on the GEE.

Variable	Estimate	Standard Error	Wald χ2	*p* Value
Intercept	−20.522	3.414	36.1	0.000 ***
Group	−1.651	0.519	10.1	0.002 **
Time	3.152	0.248	161.9	0.000 ***
Food intake	0.368	0.044	70.2	0.000 ***

** *p* < 0.01; *** *p* < 0.001.

## Data Availability

The datasets supporting the findings of this article are included within the paper and its Supplementary Materials.

## Supplementary Materials

The following supporting information can be downloaded at: https://www.mdpi.com/article/10.3390/ani15050669/s1, Figure S1: (a) Results of Venn diagram in the control group and low infection group during the 4th week of flea infection. (b) Results of Venn diagram in the control group and high infection group during the 4th week of flea infection. (c) Results of Venn diagram in the control group and low infection group during the 8th week of flea infection. (d) Results of Venn diagram in the control group and high infection group during the 8th week of flea infection.; Figure S2: (a) Dilution curves of gut microbiota samples from different groups of Brandt’s voles in the 4th week. (b) Dilution curves of gut microbiota samples from different groups of Brandt’s voles in the 8th week.; Figure S3: (a) The difference of beta diversity between the control group, the low infection group and the high infection group at the 4th week. (b) The difference of beta diversity between the control group, the low infection group and the high infection group at the 8th week. * *p* < 0.05.
