# Supplementary material for: Effects of Different Levels of Flea Infestation on Gut Microbiota of Brandt’s Voles (Lasiopodomys brandtii) in China
Source: Animals (Basel). 2025 Feb 25;15(5):669. doi: 10.3390/ani15050669 (PMC11899220; doi:10.3390/ani15050669)
Supplement: Supplementary file 1 [file animals-15-00669-s001.zip › animals-3478327-supplementary.pdf]

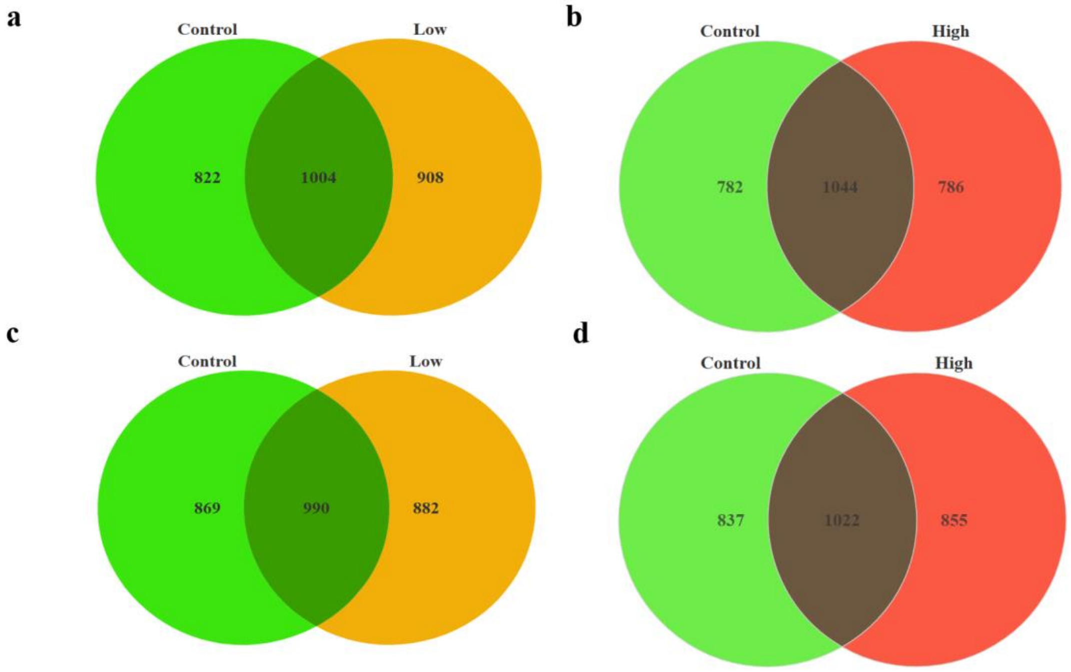

Figure S1. a. Results of Venn diagram in the control group and low infection group during the 4th week of flea infection. **b.** Results of Venn diagram in the control group and high infection group during the 4th week of flea infection. **c.** Results of Venn diagram in the control group and low infection group during the 8th week of flea infection. **d.** Results of Venn diagram in the control group and high infection group during the 8th week of flea infection.

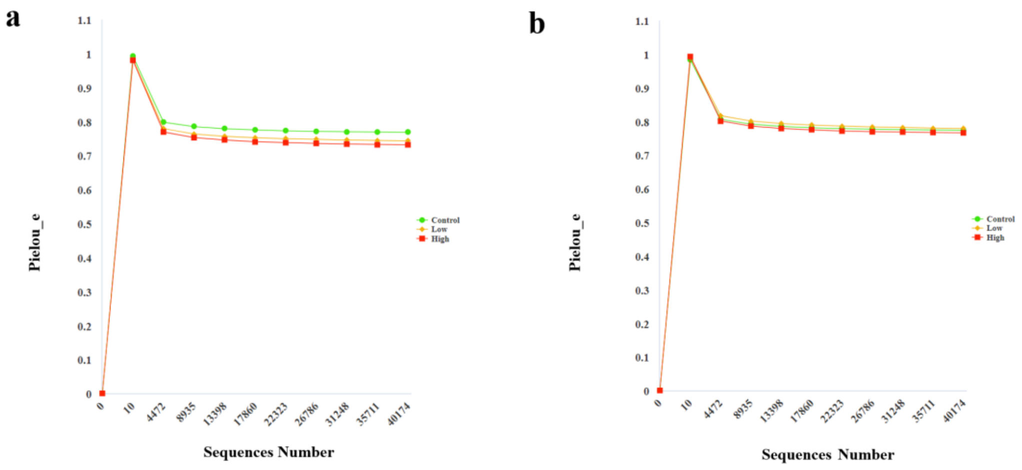

Figure S2. a. Dilution curves of gut microbiota samples from different groups of Brandt's voles in the 4th week. **b.** Dilution curves of gut microbiota samples from different groups of Brandt's voles in the 8th week.

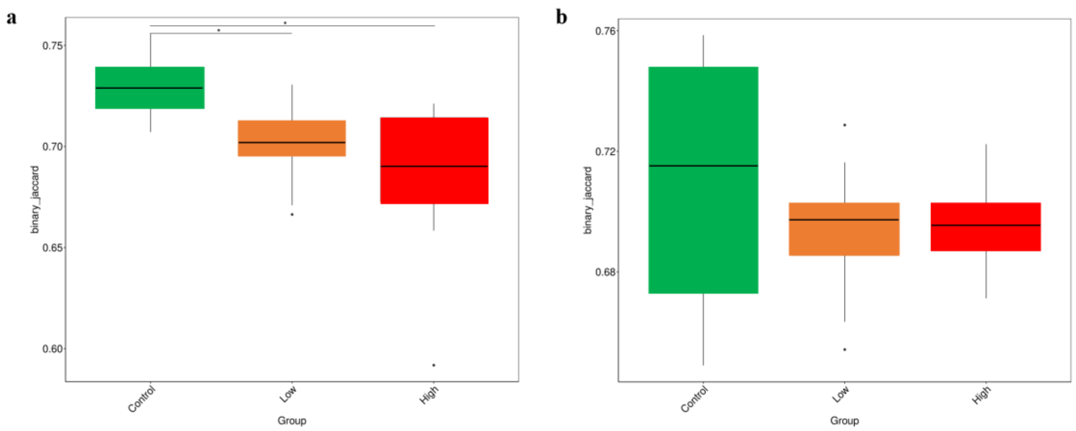

Figure S3. a. The difference of Beta diversity between the control group, the low infection group and the high infection group at the 4th week. **b.** The difference of Beta diversity between the control group, the low infection group and the high infection group at the 8th week.
